# Outcome analysis of the autologous bone grafts for reconstructing acetabular bone defects in total hip arthroplasty for developmental dysplasia of the hip

**DOI:** 10.3389/fsurg.2025.1655246

**Published:** 2025-09-11

**Authors:** Yong Zhang, Yingjie Lu, Yijian Zhang, Huilin Yang, Ming Jiang, Lixin Huang

**Affiliations:** Department of Orthopaedics, The First Affiliated Hospital of Soochow University, Soochow University, Suzhou, China

**Keywords:** developmental dysplasia of the hip (DDH), total hip arthroplasty, autologous bone grafts, acetabular bone defects, clinical outcome

## Abstract

**Objective:**

This study aimed to retrospectively investigate the short- or medium-term clinical outcomes in patients with Hartofilakidis type II developmental dysplasia of the hip (DDH) after structural bone grafting of the autologous femoral head with a non-cemented prosthesis during total hip arthroplasty (THA).

**Material and methods:**

A total of 80 patients (80 hips), including 46 men and 34 women, were enrolled. The age of the patients ranged from 44 to 61 years (mean age: 50.85 ± 9.22 years). The resected femoral head was utilized as a bone graft for the reconstruction of acetabular bone defects above the acetabulum. Preoperative and postoperative leg length discrepancies (LLD) and Harris Hip Score scores were also measured. Lastly, complication occurrence was recorded.

**Results:**

The LLD decreased from 22.13 ± 11.22 mm before surgery to 4.27 ± 2.15 mm after surgery (*P* < 0.001). The vertical distance of the hip rotation center reduced from 41.14 ± 12.17 mm before surgery to 20.76 ± 9.91 mm after surgery (*P* < 0.001). The horizontal distance of the hip rotation center diminished from 40.20 ± 13.33 mm before surgery to 22.61 ± 6.88 mm after surgery (*P* < 0.001). The HHS score increased significantly from 43.75 ± 12.67 preoperatively to 90.15 ± 8.91 at the final follow-up (*P* < 0.001). None of the patients experienced fractures during the operation, and there were no postoperative complications such as hematomas or wound infections.

**Conclusion:**

Structural bone grafting is an effective method for restoring acetabular bone volume and ensuring good acetabular prosthesis coverage in adult patients with DDH who present with intraoperative bone loss during THA.

## 1 Introduction

Developmental dysplasia of the hip (DDH) is a prevalent hip disorder (1). The incidence of DDH in adults is approximately 1.5%, with a male-to-female ratio of 1:3 (2). Late-stage DDH is often associated with secondary osteoarthritis, resulting in limited hip function, pain, and claudication (3). Patients with DDH not only have anatomical abnormalities on the acetabular side but also on the femoral head and proximal femur. In particular, the acetabulum is poorly developed and tends to become smaller and shallower with a slope-like morphology. In cases of severe dysplasia, the acetabular wall shows various degrees of bone defects (4). Furthermore, the femoral head is flattened, while the proximal femur exhibits malformed femoral anatomy with an increased anterior inclination and neck-stem angle, narrowing of the medullary cavity, and abnormal bone length (5).

In adult patients with DDH and accompanying secondary osteoarthritis and severe clinical symptoms, total hip arthroplasty (THA) is the preferred approach to restore the normal physiological function of the hip joint and eliminate pain. However, THA is more challenging in patients with severe DDH than in those with common hip osteoarthritis because of the risk of postoperative loosening and dislocation due to insufficient prosthesis coverage (6). Additionally, patients with DDH who present with secondary hip osteoarthritis requiring surgery are younger, indicating the need for long-term prosthesis survival (7). Moreover, the severity of acetabular bone deficiency compromises the initial stability of the acetabular cup and increases the risk of revision surgery (8). Therefore, appropriately managing bone defects to ensure the long-term survival of the prosthesis is vital for a successful THA.

Due to the pseudoacetabulum form in DDH, the area between the lower edge of the pseudoacetabulum and the upper edge of the true bony acetabulum is susceptible to bone loss (9). Consequently, ensuring an adequate quantity of acetabular bone to achieve satisfactory prosthesis coverage is essential. The commonly used methods to resolve bone defects and achieve good prosthetic coverage include acetabular reconstruction, structural bone grafting with autogenous or allogeneic bone, medial protrusion technique of the acetabular wall, and utilization of small prostheses (10). Autologous bone graft harvesting from the femoral head is a feasible approach to reconstruct acetabulum bone defects. This approach can not only restore the normal anatomical and mechanical relationship of the hip joint but also attain good initial stability and prosthetic coverage (11). However, autologous bone graft harvesting can also cause complications related to the grafted bone, such as resorption, collapse, displaced ossification, and osteolysis of the grafted bone, ultimately leading to the loosening of the acetabular prosthesis (12). Therefore, a series of intraoperative and postoperative strategies to promote the effective integration between the graft and host bone and to minimize complication occurrence are essential for successful surgery.

This study aimed to retrospectively investigate the short- or medium-term clinical outcomes in patients with Hartofilakidis type II DDH after structural bone grafting of the autologous femoral head with a non-cemented prosthesis during THA.

## 2 Materials and methods

### 2.1 Demographic information

From October 2012 to October 2020, the data from 100 patients (100 hips) with DDH who underwent primary THA were collected. The patient inclusion criteria encompassed no history of previous hip trauma, clinical symptoms and manifestations consistent with DDH, and the use of non-cemented prosthesis for initial THA with an autologous femoral head structural implant. Exclusion criteria comprised patients with bone cement prosthesis, severe osteoporosis, or incomplete follow-up data. After screening based on the above criteria, 80 patients (80 hips), including 46 men and 34 women, were finally selected. The age of the patients ranged from 44 to 61 years (mean age: 50.85 ± 9.22 years). According to the Hartofilakidis classification, all patients had Hartofilakidis type II DDH (Table 1).

**Table 1:** The Hartofilakidis classification of developmental dysplasia of the Hip.

Type of dysplasia	Description	Acetabular defect
Dysplasia (Type A)	The femoral head is contained in the true acetabulum	Superior segmental defect Shallowness because of osteophytes
Low dislocation (Type B)	The femoral head articulates with a false acetabulum, which partially covers the true acetabulum	Superior and anterior segmental defect Small acetabular diameter Inadequate depth Increased anteversion Lack of posterior bone stock
B1	Overlap between false and true acetabulum >50%	
B2	Overlap between false and true acetabulum <50%	
High dislocation (Type C)	Superior and posterior migration of the femoral head, which articulates with a false acetabulum that is in discontinuity with the true acetabulum	Segmental defect of the entire acetabular rim Small acetabular diameter Inadequate depth Excessive anteversion Abnormal bone stock superoposteriorly
C1	Presence of false acetabulum	
C2	Absence of false acetabulum	

### 2.2 Surgical technique

All patients were anesthetized with general anesthesia. In this procedure, the patient was instructed to lie on their healthy side, and the posterior lateral approach was employed. Initially, the skin, subcutis, broad fascia, external rotator group, and joint capsule were incised layer by layer to expose the hip joint. Intraoperative examination revealed hip joint dislocation with bone defects over the acetabulum and proximal displacement of the femoral head, which resulted in a pseudo joint with the ilium. Additionally, the femoral head was abnormally developed and severely deformed with uneven cartilage surface.

**Figure 1:**
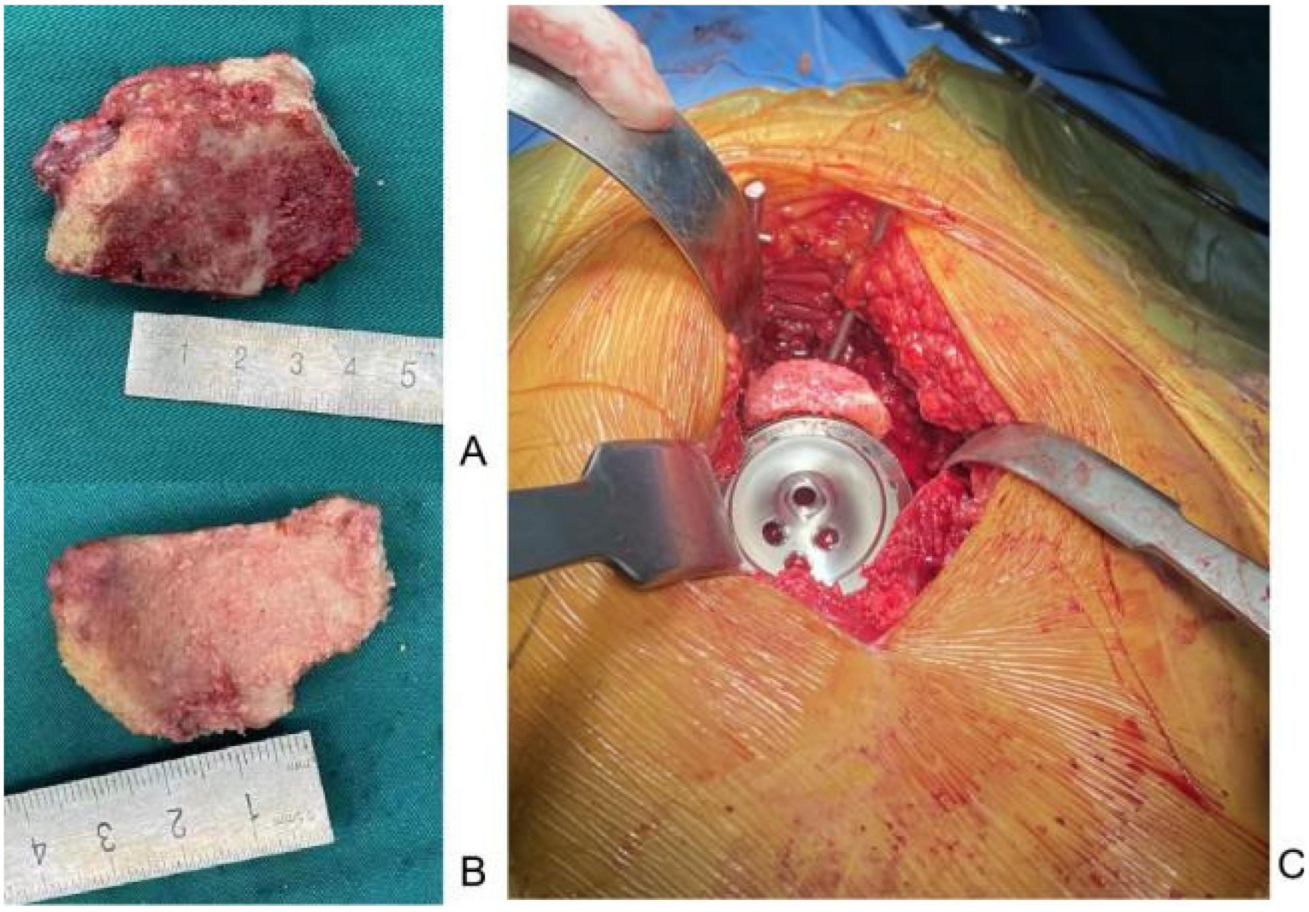
Implantation of the bone graft on the bone defect site above the acetabulum. **(A,B)** The bone block is dissected from the femoral head. **(C)** Steps of the bone grafting procedure.

After dislocating the femoral head, the femoral neck was sawn off from the base and removed. Subsequently, the cartilage layer was removed, and the resected femoral head was prepared for grafting. The acetabulum was exposed and first deepened with the smallest file, followed by a 40°–45° abduction and 10°–20° anteversion to reach the appropriate size. Furthermore, two or three cancellous bone screws were selected to secure the implant, depending on the size of the segmental defect. Any gap between the implant and the iliac bone was implanted with the autogenous pellet bone. The acetabulum underwent further filing, and the acetabular cup of appropriate size was placed and fixed with three screws (Zimmer titanium acetabular screws; Zimmer) (Figure 1).

### 2.3 Postoperative treatment

Cefazolin sodium was administered for 1 day to prevent infection, along with low molecular heparin sodium to prevent deep vein thrombosis. Patients were encouraged to start early mobilization with a walker. After hospital discharge, all patients were followed up regularly in outpatient clinics at 1, 3, and 12 months, with an annual follow-up thereafter.

### 2.4 Outcome evaluation

Preoperative and postoperative LLD and HHS scores were measured. The occurrence of complications, such as periprosthetic joint infections, periprosthetic fractures, dislocations and loosening, lower extremity venous thrombosis, nerve damage, graft bone collapse, bone resorption, and osteolysis, was also recorded. Lastly, the fusion time between the grafted bone block and ilium was also noted.

### 2.5 Statistical analysis

SPSS version 25.0 statistical package was employed to assess preoperative and postoperative HHS scores. Data such as the difference between preoperative and postoperative LLDs were analyzed using the paired-sample *t*-test and expressed as mean ± standard deviation, with a *P*-value of <0.05 statistically significant.

## 3 Results

### 3.1 Clinical outcomes

**Figure 2:**
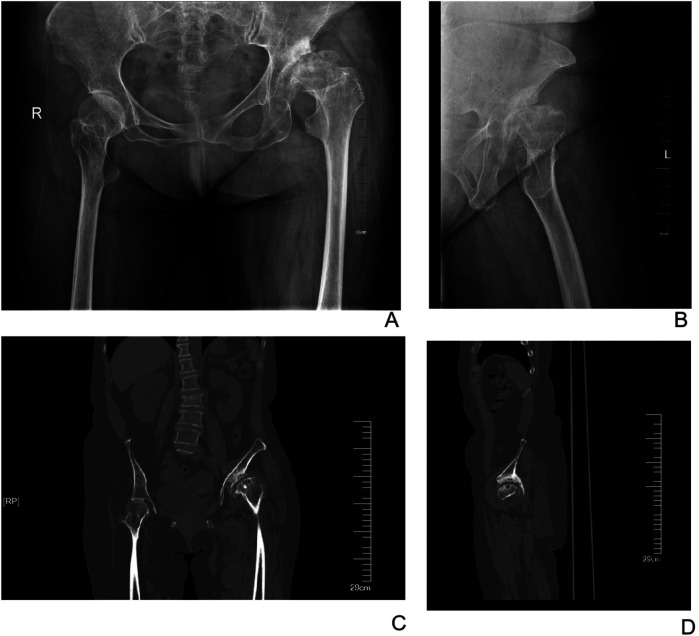
Preoperative X-ray and CT images of a 60-year-old female patient. **(A)** Anteroposterior radiograph of the hips. **(B)** Lateral radiograph of the left hip. **(C)** Anteroposterior CT image of the hips. **(D)** Lateral CT image of the left hip.

**Figure 3:**
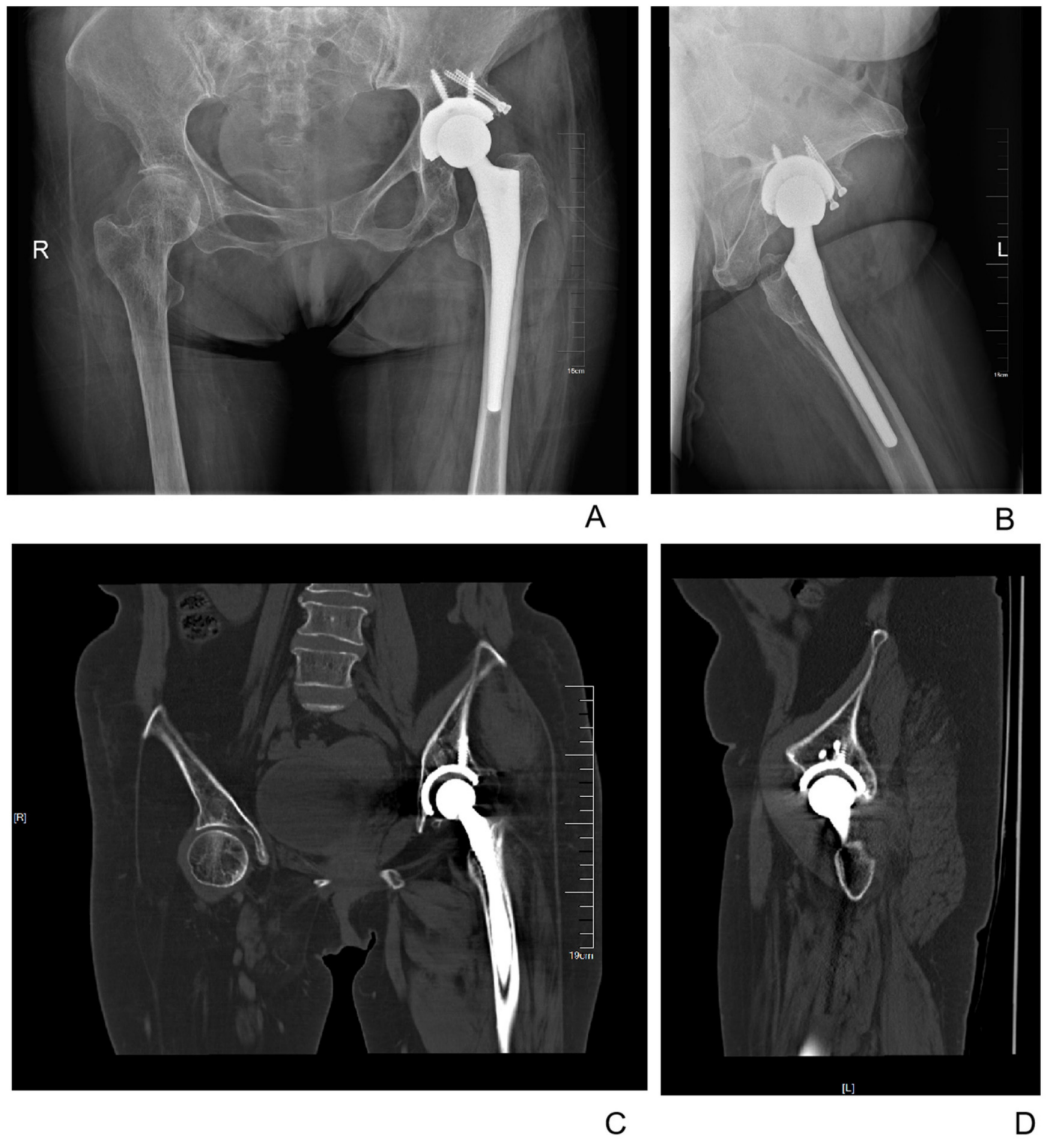
Postoperative X-ray and CT images of the female patient at 1 year after surgery. **(A)** Anteroposterior radiograph of the hips. **(B)** Lateral radiograph of the left hip. **(C)** Anteroposterior CT image of the hips. **(D)** Lateral CT image of the left hip.

The patients were followed up for a mean duration of 2.17 ± 0.23 years. The LLD decreased from 22.13 ± 11.22 mm before surgery to 4.27 ± 2.15 mm after surgery (*P* < 0.001). Furthermore, the vertical distance of the hip rotation center decreased from 41.14 ± 12.17 mm before surgery to 20.76 ± 9.91 mm after surgery (*P* < 0.001), while the horizontal distance of the hip rotation center decreased from 40.20 ± 13.33 mm before surgery to 22.61 ± 6.88 mm after surgery (*P* < 0.001). Moreover, the mean healing time for the grafted bone in-growth was 7.65 ± 4.19 months (Figures 2, 3). Finally, the HHS score improved from 43.75 ± 12.67 points preoperatively to 90.15 ± 8.91 points at the last follow-up (*P* < 0.001) (Table 2).

**Table 2:** Comparison of the outcome parameters of the study patients before and after surgery.

Outcome parameters	Before surgery	After surgery	*t*	*P*-value
Leg length discrepancies (mm)	22.13 ± 11.22	4.27 ± 2.15	13.98	<0.001
Vertical distance of the hip rotation center (mm)	41.14 ± 12.17	20.76 ± 9.91	11.61	<0.001
Horizontal distance of the hip rotation center (mm)	40.20 ± 13.33	22.61 ± 6.88	10.49	<0.001
Harris Hip Score scores (points)	43.75 ± 12.67	90.15 ± 8.91	18.23	<0.001

### 3.2 Adverse events

None of the patients had a fracture during the operation, with no postoperative hematoma, wound infection, nerve injury or heterotopic ossification. However, postoperative thrombosis of the posterior tibial vein and intermuscular venous obstruction of the affected limb were observed in one patient, which were managed with oral anticoagulant medication for 1 month. At the last follow-up, no patient showed periprosthetic osteolysis, prosthesis loosening, or socket cup loosening, and the prosthesis was fixed through bone in-growth without needing revision or other reoperation.

## 4 Discussion

Surgeons performing THA in patients with DDH usually encounter numerous technical difficulties, with acetabular reconstruction being one of them. This challenge can be further complicated by abnormal acetabular anatomy (pseudoacetabulum) and defects in the bony acetabulum. Additionally, the risk of postoperative complications and surgical failure after THA for DDH is higher than that after THA for primary osteoarthritis (13). In patients with DDH and accompanying acetabular bone loss, performing effective acetabular reconstruction and ensuring good prosthetic coverage during THA is a major challenge for orthopedic surgeons. Although several methods can be used to achieve prosthetic coverage, the most appropriate method to be used remains a highly debated topic (14).

Autologous femoral head bone graft is easy to access, does not result in rejection, and has strong osteoconductive and osteoinductive properties (15). These advantages facilitate fast bone recovery and high healing rates, leading to their frequent use as structural bone grafting materials. The structural implantation of the acetabular prosthesis in the true acetabular position not only ensures the integrity of the acetabular bone but also achieves satisfactory acetabular prosthesis coverage, which is beneficial for future revision surgery. This feature was of particular significance in our study patients because they were relatively young (average age: 40 years) at the time of surgery and were likely to require revision surgery in the future. In this study, all surgeries were performed by the same surgeon who was experienced in THA, assuring consistency in surgery quality. Furthermore, the grafted bone was fused at the final follow-up, with no signs of a late collapse. None of the patients underwent prosthetic revision. All patients in this study showed good recovery of hip function and attained satisfactory short- or medium-term clinical results. Although several researchers (16–18) have reported satisfactory early and mid-term clinical outcomes, the long-term efficacy remains debated. It is reported that a high loosening rate (46%) of the acetabular prosthesis within a follow-up period of 11.8 years postoperation (19). Structural bone grafting during THA in patients with DDH is suggested to be associated with poor long-term clinical outcomes and heightened susceptibility to complications such as prosthesis loosening and dislocation. Another study by Zahar et al. (20) found a high rate of distal prosthesis loosening in patients with DDH who underwent THA using structural implants of the femoral head. In 106 patients (115 hips), 27% of the patients had imaging translucency lines ranging from 1 to 5 mm around the acetabular prosthesis at the last follow-up, while 16% were treated with revision surgery for the aseptic loosening of the prosthesis. Moreover, Kaplan–Meier survival analysis demonstrated a 14-year prosthesis survival rate of 80%, with this rate declining sharply in the 15- to 20-year postoperative period. Therefore, we consider that our satisfactory clinical outcomes may be partially attributed to the relatively short follow-up period, and long-term patient follow-up is currently ongoing.

Currently, no universal agreement exists on the criteria for structural bone grafting in patients with DDH. Mulroy and Harris (21) suggested that at least 70% of the acetabular prosthesis should be supplied by the host bone for structural bone grafting. Further, the researchers recommended that structural bone grafting should be considered when the bone defect in the superior aspect of the acetabulum was at least 5 mm. A different study noted that acetabular prosthesis coverage by the grafted bone should not exceed 50%, thereby avoiding the excessive coverage provided by the grafted bone that might affect long-term prosthesis survival (22). Barrack et al. (23) indicated that bone grafting should be performed in cases where the acetabular prosthesis is covered by less than 75%–80% of the host bone after the molding trial of the acetabular prosthesis and at least a 90% coverage rate should be attained after bone grafting. The most common complications associated with bone grafting are bone resorption and osteolysis, which are the leading causes of prosthesis loosening. Furthermore, the most severe complication is grafted bone collapse, which is also one of the primary causes of the early loosening of the prosthesis. Heterotopic ossification is another complication associated with bone grafting, but it occurs less frequently and generally does not result in the loosening of the prosthesis. Oe et al. (24) evaluated 87 patients (101 hips) over a mean follow-up period of 11 years, and their findings showed that all patients had bone resorption of varying degrees but none with the loosening of the prosthesis. Another study found that the percentage of grafted bone resorption in the non-weight-bearing region of the acetabular prosthesis did not show statistically significant differences between patients with revision and non-revision DDH after structural bone grafting (25). Additionally, the degree of graft bone resorption and prosthesis loosening rate was significantly higher in patients having >20% of graft bone coverage than in those with graft bone coverage <20% (26). In our patients, the coverage provided by the grafted bone did not exceed 50%, with 62.9% of the patients having <25% of coverage and only 7.4% having >40% of coverage. Moreover, no patients had bone graft-related complications or prosthesis loosening or dislocation at the final follow-up. Therefore, we consider that the lower coverage provided by the grafted bone (e.g., less than 40%) may be associated with better clinical efficacy. In cases where >50% of the acetabular cup was found to be uncovered by the host bone intraoperatively, acetabular reconstruction can be performed by alternative methods, including upward or inward displacement.

This study has several limitations that restrict the generalizability of the conclusion. For example, this study was a retrospective investigation with a possible selection bias. Additionally, the follow-up period was limited, and no control group was included. Moreover, only x-ray images were used for the imaging criteria-based assessment of fusion and resorption of the grafted bone in the follow-up. However, CT scanning, which can provide further details of the grafted bone, was not employed. Therefore, the long-term status of the grafted bone and the extent of its impact on the acetabular prosthesis warrant further investigation.

## 5 Conclusions

Structural bone grafting is an effective method for restoring acetabular bone volume and ensuring good acetabular prosthesis coverage in adult patients with DDH who present with intraoperative bone loss during THA. However, this strategy is also associated with the risk of graft-related complications such as resorption, collapse, and displaced ossification of the grafted bone. Therefore, surgeons should be familiar with the indications for structural bone grafting as well as the proper handling and fixation of the grafted bone and the selection of the appropriate prosthesis to potentially improve the long-term survival rate of the prosthesis and achieve satisfactory clinical outcomes.

## Data Availability

The raw data supporting the conclusions of this article will be made available by the authors, without undue reservation.
